# Nicotine and Cognition in Cognitively Normal Older Adults

**DOI:** 10.3389/fnagi.2021.640674

**Published:** 2021-05-05

**Authors:** Olivia Nop, Anna Senft Miller, Hannah Culver, Jenna Makarewicz, Julie A. Dumas

**Affiliations:** Department of Psychiatry, University of Vermont, Burlington, VT, United States

**Keywords:** cognitive aging, nicotine, memory, cognition, normal aging

## Abstract

The cholinergic system has been shown to be the primary neurotransmitter system which is responsible for the cognitive symptoms associated with dementia; its role in healthy non-demented older adults remains a gap in the literature. Understanding the effects of age-related functional changes on the nicotinic system will address this knowledge gap. As the older adult population grows and hence the importance of understanding cognitive changes that impact functional abilities and everyday life. In this article we examine the benefits of using nicotine as a method for improving cognition in non-demented healthy older adults which may have the potential for slowing neurodegeneration in aging. Furthermore, we discuss how nicotine can play a crucial role in maintaining cognitive abilities throughout normal cognitive aging.

## Introduction

While Alzheimer’s disease (AD) and dementia continue to affect an increasing number of the older adult population, a majority of older adults will not be diagnosed with dementia. An Institute of Medicine report (Blazer et al., 2015) characterized the notable changes in cognition during aging that are not dementia yet still affect every day functioning and quality of life for older adults. There is a large body of research from cognitive psychology of aging demonstrating changes in processes like processing speed (Salthouse, 1996), attention (McDonough et al., 2019), working memory (Gazzaley et al., 2005), and executive functioning (Silver et al., 2011) that do not rise to the level of a dementia diagnosis yet have an impact on older adult lifestyles. There is also a growing literature (Phillips, 2017) on non-pharmacological attempts to improve cognition in aging including exercise (Falck et al., 2019), cognitive training (Toril et al., 2014), diet (Morris et al., 2015), and social interaction (Evans et al., 2018) and each method has its benefits and challenges. We suggest a pharmacological approach to influence cognition in older adults using the cholinergic agonist nicotine. If nicotine is shown to be beneficial for cognition, it can be combined with any of the non-pharmacological approaches mentioned above to improve quality of life for older adults. Research has suggested that nicotine may benefit cognitive functioning in older age (Gandelman et al., 2019), as nicotine has been shown to improve cognition in older adults with mild cognitive impairment (MCI; Newhouse et al., 2012), age associated memory impairment (White and Levin, 2004), and AD (White and Levin, 1999). However, there is limited knowledge about nicotine’s effects on normal cognitive aging; there have been few studies investigating the relationship between stimulation of nicotinic acetylcholine receptors (nAChRs) with nicotine and its effects on cognition in healthy older adults (Min et al., 2001; Niemegeers et al., 2014). We present information that offers potential mechanisms for how nicotine may play an important role in slowing normal cognitive changes in aging and suggest future studies to fill in the information gaps in the current knowledge.

Below, we first describe normal cognitive aging. Then, we describe the cholinergic system and its role in normal and pathological cognition. Next, we describe the literature on nicotinic stimulation and cognition in adults with and without cognitive impairment. Finally, we offer suggestions for future studies to examine the role of nAChR stimulation for enhancing cognition in healthy older adults.

## Cognitive Changes in Normal Aging

The changes that occur in brain structure and function as a result of normal aging are referred to as cognitive aging and have been an area of focus in cognitive psychology for over 50 years (for a review, see Anderson and Craik, 2017). While some cognitive abilities grow stronger or remain unaffected by age, such as vocabulary and general knowledge, skills that require more effortful processing or have a high attentional load deteriorate with age (Harada et al., 2013). Furthermore, fluid intelligence, including problem solving, attention, and memory, noticeably decline as aging progresses (Labouvie-Vief, 2003; Salthouse, 2012). This decline has real life implications, including, for example, decreased ability to live independently, a decreased ability to manage finances, and an increased susceptibility to financial scams (Blazer et al., 2015). By understanding the neurobiological mechanisms by which higher cognitive processes decline with age, there may be strategies available to mitigate the effects of aging on brain functioning.

## The Cholinergic System, Cognition, and Dementia

Studies have shown that normal cognitive aging is characterized by difficulties in effortful processing (Craik and Salthouse, 2000). The neurobiology underlying effort-demanding cognitive functioning has been linked to the cholinergic system (Warburton and Rusted, 1993). Warburton and Rusted (1993) demonstrated that the cholinergic system is involved in the limited executive resources that allow for successful performance on attention and memory tests. Specifically, they showed that stimulating the cholinergic system with nicotine improved working memory in non-smoking younger adults (Warburton and Rusted, 1993). Preclinical studies have shown that the cholinergic system supports lower level cognitive processes like attention and perception (Sarter et al., 2005), which are known to decline in normal aging in humans (Verhaeghen et al., 1993). Thus, we and others have proposed that age-related changes in cholinergic system functioning are responsible for the age related decrease in attention and perception (Dumas and Newhouse, 2011). However, further work is needed to fully elucidate the role of the changes in cholinergic functioning and their effects on cognition in normal aging.

Much of what is known about the cholinergic system and cognition in older adults is from studies of patients with dementia. Earlier research has shown that the cholinergic system is the primary neurotransmitter system responsible for cognitive symptoms in dementia (Bartus et al., 1982). The cholinergic hypothesis of geriatric memory dysfunction, proposed by Bartus et al. (1982), hypothesized that functional disturbances in cholinergic activity occur in the brains of patients with dementia. These disturbances consequently play a role in memory loss and related cognitive problems. This hypothesis has been supported by the finding that cholinesterase inhibitors positively affect cognition in patients with AD (Hansen et al., 2008). A large volume of clinical research on cholinergic agents has followed since the initial proposal of the cholinergic hypothesis. Although the overall clinical effects are limited, the cognitive enhancers that modulate cholinergic functioning remain the most widely used medications approved for use in AD.

Nicotine is a direct agonist at the nAChR (Nees, 2015) and has been shown to have cognitive enhancing properties across a wide range patients with psychiatric disorders (e.g., Potter and Newhouse, 2008) and memory impairment (Newhouse et al., 2012). Studies of smokers, abstinent smokers, and non-smokers in younger adults have shown that the nicotinic system is involved in attention and memory (Warburton and Rusted, 1993), and nicotinic stimulation can modulate functioning in cognitive domains such as response inhibition, emotion, cue reactivity and reward processing (Heishman et al., 2010; Nees, 2015). A thorough review of this literature on the cognitive effects of nicotine in smokers, abstinent smokers, and non-smokers is beyond the scope of this article, which is focused on normal cognitive aging, and is reviewed elsewhere (i.e., Campos et al., 2016). Many of these studies have demonstrated positive effects of nicotine for cognition with acute dosing, and safety and efficacy have also been seen in studies with chronic nicotine treatment (Newhouse et al., 2012). Overall, there appear to be positive effects of nicotine for cognition across a wide range of subject and patient populations. Next, we examine the ability of nicotine to affect cognition in older adults.

## Nicotine, Cognition, and Aging

**Figure 1:**
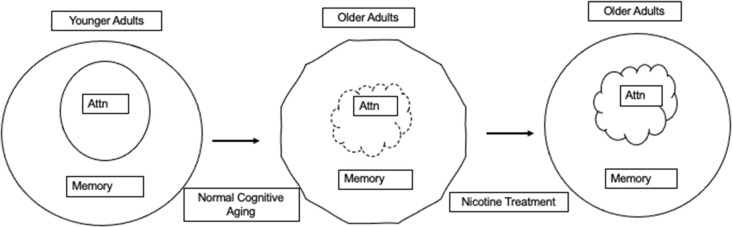
Younger adult cognition is represented with attention supporting normal memory functioning. In normal cognitive aging there is a break down in lower level attentional processes that has the potential to affect memory. We and others hypothesize that nicotinic stimulation can restore nicotinic system functioning resulting in attentional improvements that can also improve memory.

In dementia, structural changes in the nicotinic system appear to be related to cognitive dysfunction (Picciotto and Zoli, 2002). However, there is no evidence for structural changes in the nicotinic system in those who are aging non-pathologically, although cognitive decline still occurs (Decker, 1987; Blazer et al., 2015). Because the structural integrity of the nicotinic system is not compromised in normal aging, it implies that a manipulation aimed at enhancing nicotinic functioning may lessen the cognitive symptoms of aging that are normally supported by the nicotinic system because the receptors remain available and can potentially be manipulated to enhance or maintain the role of the nicotinic system in cognition (see Figure 1).

Earlier studies have shown that chronic nicotine treatment was beneficial for cognitive functioning in older adults with memory impairment. Nicotine treatment improved attention in older adults with MCI (Newhouse et al., 2012), age associated memory impairment (White and Levin, 2004), and AD (White and Levin, 1999). While nicotine treatment was safe and effective in these studies, there were differences in the length of dosing. Newhouse et al. (2012) gave transdermal nicotine vs. placebo for 6 months while the studies by White and Levin (1999, 2004) used a 4-week, placebo controlled, double blind, cross over treatment phase. These three studies primarily examined safety of nicotine treatment and used the continuous performance test (CPT) to measure efficacy on cognition showing nicotine improved attention in the older adults in each study. Longer treatment studies are needed to evaluate the continued safety and efficacy of nicotine treatment for older adults with memory impairment. Although more studies are needed, nicotine is one of the few medications that has been shown to have any effectiveness for improving cognition in pathological aging. Perhaps one way to further understand the potential benefits or mechanisms of nicotine’s effects on cognition is to employ imaging methods during nicotine administration. There are some recent studies in older adults that show promising effects of nicotine action in the aging brain.

The development of nicotinic radioligands has enabled the use of polyethylene terephthalate (PET) imaging allowing researchers to examine the structure and function of the nicotinic system and how it is affected by pathological and normal aging. Earlier studies used PET to examine the correlation between acetylcholinesterase (AChE) activity and the cognitive decline in AD as well as MCI (Shinotoh et al., 2000; Herholz et al., 2005). Low levels of AChE activity were found to precede dementia and further reduce during the progression of AD (Shinotoh et al., 2000; Herholz et al., 2005). More recently, Richter et al. (2014) examined the cerebral cholinergic activity in memory-relevant brain areas in healthy adults. This study used N-methyl-4-piperidyl acetate (MP4A), a radiolabeled cholinesterase substrate. The study showed a negative relationship between periventricular white matter lesions and cerebral AChE activity (Bohnen et al., 2005) which suggests that cholinergic decline accompanies structural brain changes in aging.

In a more recent PET study that used a nicotinic receptor ligand, Sultzer et al. (2017) showed that lower nAChR binding in the thalamus and hippocampus was correlated with slower processing speed in healthy older adults (Sultzer et al., 2017). Slower processing speed is one of the major hallmarks of normal cognitive aging (Salthouse, 1996). Thus, these PET data were the first to find a neurobiological substrate for the cognitive slowing that is nearly ubiquitous in normal aging. Whether nicotinic stimulation can alter this cognitive slowing is yet to be determined.

At present, the direct effect of nicotine on cognition in healthy older adults is under examined and no studies have examined long-term effects of nicotine for cognition in healthy older adults. The studies used acute doses and found variable effects of nicotine. Min et al. (2001) conducted a randomized, double-blind study of 5 mg of transdermal nicotine or a placebo patch to healthy participants aged 60–69 years. The effects of nicotine on memory and attention were quantified using the Short Blessed Test, Rey-Kim Memory Test, and Digit Span Test (Min et al., 2001). Nicotine blood levels were measured after the cognitive testing before patch removal. Subjects were selected for the study who did not have cognitive impairment. First, the results showed no effect of nicotine on the Short Blessed Test which was used as the measure of cognitive impairment in this sample of Korean older adults. The subjects were then grouped into three groups according to plasma nicotine levels of low, medium, and high values. The results showed that adults in the low nicotine plasma group had improved short-term verbal memory performance compared to pre-drug testing. In addition, higher nicotine levels were associated with a higher slope measure representing a faster learning rate. Thus, there was a signal for nicotine improving performance in the older adults in this sample (Min et al., 2001). This study showed that there was an optimal range for observing the benefits of nicotine on cognition and that range may depend on a number of variables from individual subject characteristics to the cognitive task components being examined.

Niemegeers et al. (2014) also examined the effects of acute nicotine on cognition in older adults and also tested younger adults as a comparison group. They used acute doses of 1 mg, 2 mg, and placebo nicotine nasal spray in younger (aged 18–30 years) and older (aged 60–75 years) adults. The cognitive battery included assessments of attention, working memory, visual memory, information processing speed, psychomotor function, stereotypy, and emotional recognition. The results showed that older adults performed worse than younger adults after placebo on the tests of psychomotor speed and information processing speed. Nicotine had no effect on performance in younger adults. In older adults, nicotine resulted in reduced accuracy in working memory and visual memory. Secondary analyses showed that within each age group, nicotine did improve cognition in those participants with lowest level of performance in the younger and older age groups. However, nicotine impaired performance in the group who performed the best under the placebo condition. Niemegeers et al. (2014) proposed that the benefits of nicotine were dependent upon baseline performance and their data showed those with lower performance were more likely to see improvements in cognition. These data showed evidence for an inverted-U shaped relationship between nicotine and performance with lower levels of nicotine providing a greater benefit and higher levels representing overstimulation and impaired performance in particular for older adults (Niemegeers et al., 2014). Thus, the dose, method of administration, and individual difference factors all contribute to the ability of nicotine to improve cognition in older adults, but what combinations are optimal for improvements in cognition needs further study.

The results from the Min et al. (2001) and Niemegeers et al. (2014) showed that the effects of nicotine on cognition in older adults were variable and dependent upon baseline cognitive ability and nicotine blood levels. Further studies with larger Ns are needed to determine the conditions under which nicotine can be beneficial and for whom. Neither earlier study was large enough to examine individual differences in factors such as BMI and drug metabolism and factors such as these should be evaluated. However, studies in older adults with cognitive impairment showed that chronic nicotine administration revealed a cognitive benefit. Thus, there are indications that nicotine treatment may improve cognition in older adults, but the safety and efficacy of any chronic nicotine dosing in healthy older adults needs further study.

## Suggestions for Future Studies

In 2020, 5.8 million Americans were diagnosed with AD and risk of AD increases dramatically with age. Additionally, the majority of the older adult population who do not have dementia may still experience cognitive changes that are not classified as pathological aging but have important impacts on everyday functioning. Therefore, there would be a great benefit to society in discovering a safe and effective cognitive enhancer that has the potential to assist healthy older adults in living independently into older ages. If stimulation of the nicotinic system with agonists such as nicotine can improve and sustain cognition in older adults, it has the potential to be used in combination with non-pharmacological methods. These combinations can be used to slow cognitive changes and as preventative care for people with an increased risk for dementia. Many questions remain about how nicotine treatment in normal cognitive aging would proceed including the length of treatment, the dose of nicotine, recommendations for those who are already smokers, guidelines based on AD risk factors like APOE genotype, and many others. While data from studies of psychiatric and memory-impaired subject populations indicate nicotine may be helpful for cognitive symptoms, further study examining the benefits of nicotine in normal aging is warranted to fill this gap in the literature as well as to verify whether nicotine should use used as a pharmacologic tool to slow cognitive aging.

## Author Contributions

All authors helped conceptualize the manuscript idea. All authors contributed to the article and approved the submitted version.

## Conflict of Interest

The authors declare that the research was conducted in the absence of any commercial or financial relationships that could be construed as a potential conflict of interest.
